# Abnormal degree centrality values as a potential imaging biomarker for major depressive disorder: A resting-state functional magnetic resonance imaging study and support vector machine analysis

**DOI:** 10.3389/fpsyt.2022.960294

**Published:** 2022-09-06

**Authors:** Hang Lin, Xi Xiang, Junli Huang, Shihong Xiong, Hongwei Ren, Yujun Gao

**Affiliations:** ^1^Department of Psychiatry, Tianyou Hospital Affiliated to Wuhan University of Science and Technology, Wuhan, China; ^2^Key Laboratory of Occupational Hazards and Identification, Wuhan University of Science and Technology, Wuhan, China; ^3^Department of Spine and Orthopedics, Tianyou Hospital Affiliated to Wuhan University of Science and Technology, Wuhan, China; ^4^Department of Medical Imaging, Tianyou Hospital Affiliated to Wuhan University of Science and Technology, Wuhan, China; ^5^Department of Nephrology, Tianyou Hospital Affiliated to Wuhan University of Science and Technology, Wuhan, China; ^6^Department of Psychiatry, Renmin Hospital of Wuhan University, Wuhan, China

**Keywords:** major depressive disorder, rest state fMRI, degree centrality, support vector machine, biomarker

## Abstract

**Objective:**

Previous studies have revealed abnormal degree centrality (DC) in the structural and functional networks in the brains of patients with major depressive disorder (MDD). There are no existing reports on the DC analysis method combined with the support vector machine (SVM) to distinguish patients with MDD from healthy controls (HCs). Here, the researchers elucidated the variations in DC values in brain regions of MDD patients and provided imaging bases for clinical diagnosis.

**Methods:**

Patients with MDD (*N* = 198) and HCs (*n* = 234) were scanned using resting-state functional magnetic resonance imaging (rs-fMRI). DC and SVM were applied to analyze imaging data.

**Results:**

Compared with HCs, MDD patients displayed elevated DC values in the vermis, left anterior cerebellar lobe, hippocampus, and caudate, and depreciated DC values in the left posterior cerebellar lobe, left insula, and right caudate. As per the results of the SVM analysis, DC values in the left anterior cerebellar lobe and right caudate could distinguish MDD from HCs with accuracy, sensitivity, and specificity of 87.71% (353/432), 84.85% (168/198), and 79.06% (185/234), respectively. Our analysis did not reveal any significant correlation among the DC value and the disease duration or symptom severity in patients with MDD.

**Conclusion:**

Our study demonstrated abnormal DC patterns in patients with MDD. Aberrant DC values in the left anterior cerebellar lobe and right caudate could be presented as potential imaging biomarkers for the diagnosis of MDD.

## Highlights

– The support vector machine (SVM) was used to differentiate between major depressive disorder (MDD) and healthy controls.
– Patients with MDD reported abnormalities in brain scans.
– The left cerebellum anterior and right caudate were the potential specific biological imaging markers for patients with MDD.

## Introduction

Major depressive disorder (MDD), a well-researched psychiatric disorder, occurs with a high rate of disability, which presents a primary cause of the economic burden throughout the world (1). As per the World Health Organization report in 2017, about 322 million people suffer from depression, ranking second in the world’s disease burden and growing to the largest in 2030 (2). Despite the tremendous burden brought by MDD, the existing studies have not found useful diagnostic markers.

Previous neurological imaging studies have implicated functional and structural aberrations in patients with MDD. However, different neuroimaging features between various investigations have been identified. Structural brain imaging studies show the lesser gray-matter volume in the insula and various subcortical and medial temporal regions, including the left sides of the caudate, hippocampus, parahippocampal gyrus, and cerebellar areas of patients with MDD (3). Also, hippocampal structural reductions have been tied explicitly to MDD illness progression (4). The common analysis methods of functional brain imaging include regional homogeneity (ReHo), low-frequency fluctuation (ALFF), and functional connectivity (FC). Previous research found elevated FC values in the bilateral parietal and left occipital regions (5) and depreciated resting-state functional connectivity (rsFC) between the left superior frontal gyrus and hippocampus (6). Besides, MDD patients showed elevated ALFF in the right superior frontal gyrus (SFG) and depreciated ALFF in the bilateral precuneus, bilateral cerebellum, and left occipital cortex (7). Geng et al. found elevated ReHo in the bilateral parahippocampal gyrus and left lingual gyrus but depreciated in the right middle frontal gyrus in patients with depressive disorders who showed somatic symptoms (8). These studies revealed abnormalities in brain function in patients with MDD. However, ALLF and ReHo reflect local brain activity and do not show the functional connection between different brain regions. When abnormal FC exists between two brain regions, the FC analysis method is challenging to determine the anomalous brain region. Our study aimed to use the technique of degree centrality (DC) to detect resting state functional connections in patients with MDD.

DC takes into account the relationship of a given region with that of the entire functional connectome and not just its relation to individual areas or separate more significant components (9). The DC analysis method completes functional connectivity across the brain and shows brain regions with abnormal signals. Previous studies have demonstrated the applicability of the DC analysis method to elucidate abnormalities in brain networks in different psychiatric and neurologic disorders. For instance, elevated levels of DC were identified in the schizophrenia group in the right inferior parietal lobule/angular gyrus relative to the HCs (10). However, the tinnitus patients showed elevated DC in the left inferior parietal gyrus and depreciated DC in the left precuneus within the dorsal attention network (11). This lack of consistency could be attributed to variations in disease characteristics or symptoms. It could also suggest aberrant brain activity that could be reflected in modifications in DC values. In addition, the abnormality of the DC value was also found in the research on MDD (12, 13). However, few researchers have combined DC and support vector machine (SVM) methods in studies of MDD.

SVM can be used as a rigorous machine learning methodology working by constructing a hyperplane that separates the samples based on the maximum margin approach (14). It could be used to predict psychosis based on neuroanatomical biomarkers. Compared with other machine learning methods such as artificial neural networks, SVM can successfully solve high dimensional and local minimum problems with better generalization. Therefore, SVM is widely used to distinguish patients with epilepsy (15), Tourette syndrome (16), schizophrenia (17), and MDD (18) from HCs. This study investigated DC values in patients with MDD, studied brain areas with modified DC values, and described the regions as probable neurological imaging markers *via* the SVM method. We hypothesized that DC in patients with MDD might be abnormal, and SVM might screen out the most valuable brain regions for diagnosing MDD.

## Materials and methods

### Subjects

In this study, one hundred ninety-eight patients with MDD were selected from the Department of Psychiatry at the Tianyou Hospital, affiliated with the Wuhan University of Science and Technology. We applied a 17-item Hamilton Rating Scale for Depression (HRSD-17) to understand the severity of the depressive state of a patient, as per the Diagnostic and Statistical Manual of Mental Disorders, fourth edition (DSM-IV). Two psychiatrists completed the diagnosis. Two hundred thirty-four healthy controls (HCs) matched with the experimental group, including age, gender, and years of education, were recruited. HCs were repeatedly screened to exclude any background of mental illness.

The exclusion criteria for subjects were as follows: (1) subjects showing symptoms complying with the symptoms of other psychiatric disorders meeting DSM-IV diagnostic criteria, such as schizophrenia, anxiety disorders, and bipolar disorder; (2) past or present significant physical diseases, such as cardiovascular disease or diabetes; (3) a history of head injury or other neurologic diseases; (4) pregnancy; (5) contraindications for MRI scan. (6) Left-handedness.

The ethics committee of Tianyou Hospital, affiliated with the Wuhan University of science and technology, sanctioned the study protocol. Written informed consent was obtained from all study subjects.

### Image acquisition

MRI scans were obtained using the Ingenia 3.0 T (Philips, Amsterdam, The Netherlands). The scanner noise was minimized using earplugs; the head motion was reduced using foam padding. Patients were required to stay conscious and relax. High-resolution 3D T1-weighted structural images were acquired with following parameters: echo time (TE) = 3.2 ms; repetition time (TR) = 7.2 ms; field of view (FOV) = 256 mm × 256 mm; flip angle (FA) = 7°. RS blood-oxygen-level-dependent (BOLD) fMRI data were obtained with the following parameters: FOV = 220 mm × 220 mm; TE = 30 ms; TR = 2021 ms; FA = 90°; slice thickness = 3.5 mm.

### Imaging preprocessing

Resting state data were preprocessed using DPABI^1^ on MATLAB 2013b. The first five time points were discarded until the subjects became accustomed to the scanner’s noise. The remaining images were slice-time-corrected and spatially realigned for head motion. We estimated the translation volume in each direction and individual axial rotation to elucidate head motion parameters. The BOLD data for each subject were within the defined motion threshold (The translation threshold was set to ± 2 mm, while the rotation threshold was limited to ± 2°). Spatial normalization of the functional images was done using echo-planar imaging sequence templates. We performed linear detrending and filtering (0.01–0.08 Hz) of all images to reduce the high-pitch respiratory and cardiac noises. We performed regression analysis to remove the white matter signal, the head motion parameters, and the cerebrospinal fluid signal, followed by removing the linear trends.

### Degree centrality analysis

DC is a theory-based graph method to elucidate the connection degree between each node and other nodes in the network. The REST^2^ software calculated the voxel-based DC value of the whole brain gray matter. We calculated the Pearson correlation coefficient between the bold time processes of all voxel pairs. For a given voxel, DC is calculated as the sum of positive functional connections between this voxel and all other voxels in the gray matter above the threshold of 0.25 (19), and then the individual voxelwise DC was converted into a Z-score map. Finally, the resulting DC maps were spatially smoothed with a 6-mm full width at half-maximum (FWHM) Gaussian kernel (detailed information can be found in Supplementary Material).

### Statistical analysis

SPSS v22.0 software was used to compare clinical data and demographic data. The age, HRSD score, and years of education of the two groups were compared by two-sample *t*-test, and the gender distribution was analyzed by chi-square test. To explore the difference of DC between MDD patients and HCs, a voxel-by-voxel two-sample *t*-test was performed. The significance threshold was set at *p* < 0.01 and Gaussian random field theory (GRF) was employed to correct multiple comparisons through using REST at *p* < 0.05 (voxel significance: *p* < 0.001, cluster significance: *p* < 0.05). The abovementioned *t*-tests were performed with gender, age, and years of education as covariates as these factors may confound the results (2, 12).

### Classification analyses

The SVM method has been diffusely applied in disease diagnosis for neuropsychosis (15, 18, 20). Running the LIBSVM^3^ software package in MATLAB, the SVM methodology was used to test the sensitivity, accuracy, and effectiveness of using DC values identified in abnormal brain regions to distinguish MDD from HCs (detailed information can be found in Supplementary Material).

## Results

### Clinical characteristics of major depressive disorder and healthy controls

The clinical data of MDD and HCs are shown in Table 1. No significant intergroup differences were observed in age, gender, and education (*p* > 0.01). The HRSD scores of MDD groups were substantially higher than those of the HCs group (*p* < 0.01).

**TABLE 1:** Demographic and clinical characteristics.

Characteristics	Patients (*n* = 198)	HCs (*n* = 234)	*P-values*
Gender (men/women)	198 (102/96)	234 (130/104)	0.401
Age, years	28.01 ± 7.442	27.87 ± 6.492	0.832
Years of education, years	12.05 ± 3.325	12.55 ± 2.931	0.100
HRSD-17	23.63 ± 2.547		

The p-value for the gender distribution was obtained by the Chi-square test. The other p-values were obtained by two sample t-tests. HCs, healthy controls; HRSD-17, 17-item Hamilton Rating Scale for Depression.

### Degree centrality analysis

**FIGURE 1:**
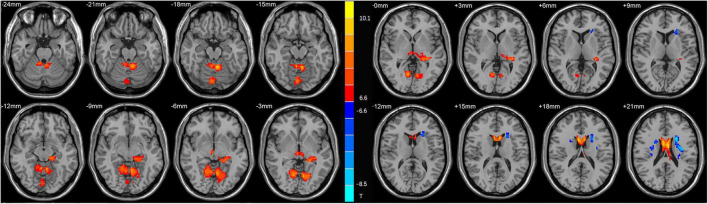
Statistical map depicts higher and lower degree centrality (DC) of major depressive disorder (MDD) patients compared with healthy controls (HCs). The threshold was set at *p* < 0.01. Blue denotes lower DC, and red denotes higher DC. The color bar indicates *t*-values from the two-sample *t*-test.

As shown in Table 2 and Figure 1, the main results reported were based on the DC analysis. According to a two-sample *t*-test, compared with HCs, MDD displayed elevated DC in the left anterior cerebellar lobe, vermis, left hippocampus, left caudate, and depreciated DC in the left posterior cerebellar lobe, left insula, and right caudate.

**TABLE 2:** Significant DC differences across groups.

Cluster location	Peak (MNI)			Number of voxels	*t*-value
	X	Y	Z		
Left cerebellum posterior	–21	–39	–57	40	–8.6051
Left cerebellum anterior	–6	–54	–15	515	9.6958
Vermis	3	–84	–15	78	8.5139
Left hippocampus	–33	–33	3	179	8.8014
Left insula	–24	9	24	246	–8.9089
Left caudate	–6	–3	24	206	10.841
Right caudate	24	0	27	118	–7.8289

DC, degree centrality; MNI, Montreal Neurological Institute.

### Discriminating patients with major depressive disorder from healthy controls

**FIGURE 2:**
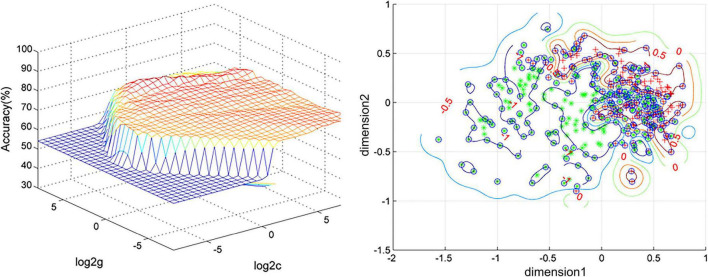
Visualization of classifications through support vector machine (SVM) using the combination of the degree centrality (DC) values in the left cerebellum anterior and right caudate. Left: SVM parameter result of 3D view. Right: Dimension 1 and dimension 2 represent the DC values in the left cerebellum anterior and right caudate, respectively. Red crosses represent healthy controls (HCs), and green crosses represent patients with major depressive disorder (MDD).

We use SVM to distinguish MDD and HCs. As per the results, DC values in the left cerebellar anterior lobe and right caudate can distinguish MDD from healthy subjects with high accuracy, specificity, and sensitivity of 87.71% (353/432), 84.85% (168/198), and 79.06% (185/234), respectively (Figure 2).

## Discussion

The current diagnosis of MDD relies on the depression scale and the clinicians’ subjective analysis, thus, lacking objective imaging methods. We investigated brain network node neutrality changes between MDD and HCs at rest. We found that compared with HCs, the DC values of patients with MDD in the left cerebellum posterior, left insula, and right caudate nucleus depreciated. In contrast, the DC values of the left cerebellum anterior, vermis, left hippocampus, and left caudate were elevated. In addition, the combination of DC values of the left cerebellum anterior and right caudate nucleus, as per the SVM analysis, might be used as potential imaging markers for the diagnosis of MDD.

More attention has been paid to cerebellar function in motor control, ignoring unexplained cognitive or neuropsychiatric phenomena. But studies in recent years have confirmed that the cerebellum participates in cognitive and emotional regulation through polysynaptic connections with different brain functional regions (21–24). For example, reduced lobular VIIA-vmPFC connectivity was significantly associated with impaired verbal working memory performance in depressed patients (25). In emotion regulation, MDD patients needed more vital expression to identify relaxing/positive emotions and sad emotions through slight expression, which implied that patients with MDD exhibited a bias in mood-congruence in facial expression procession. The posterior cerebellar lobe was confirmed to be involved in this dependent process. In addition to emotional processing, some studies have shown that the posterior part of crus I and II of lobule VIIA and lobule IX are associated with DMN (26–28). There appears to be a strong correlation between MDD and DMN, a crucial aspect of the neurobiology of MDD, specifically for episodic memory retrieval, self-referential activity, intrinsic attention allocation, and emotional behavior modulation-induced symptoms (29). This further indicates that the cerebellum is critical for the pathophysiological process of MDD. It is noteworthy that most of these experimental results were in the posterior cerebellum. Still, we found abnormal DC values in the anterior cerebellar lobe of patients with MDD by combining them with SVM. This might have been related to the current course of treatment of the patients (30).

In addition, we also found the abnormal DC value in vermis in patients with MDD. Vermis has been found to play a regulatory role on the subcortical nodes of the salience networks (SN) (28). SN plays a crucial role in the bottom-up identification of relevant events to allow for the application of appropriate resources when relevant events are identified (31) and also alters the central executive networks and the default mode. This ability to switch has been confirmed to be impaired in patients with MDD. Therefore, we can infer that vermis regulates the cognitive and emotional function in patients with MDD.

The hippocampus is an important region involved in memory and cognitive function (32), composed of different subregions interacting with varying areas of the brain, resulting in a neuro-anatomical network of emotion regulation and cognitive processing (33). For instance, the hippocampal volume of patients with MDD depreciated substantially (34, 35) and elevated after electroconvulsive therapy (ECT) relative to HCs (36). Moreover, the less posterior-DMN-hippocampal connectivity was correlated to elevated cognitive activity and rumination in MDD (37). Hao et al. also found that different subareas of the hippocampus (including the subiculum and dentate gyrus) were substantially related to MDD (32). As per our results, an elevated DC value was identified in the left hippocampus in patients with MDD, which confirmed that the hippocampus was involved in the pathogenesis of MDD.

In addition, a decrease in the DC value of the left insula was observed in MDD. The insula is a critical node for integrating external emotional stimuli (38). In previous studies of MDD, the functional activity and connectivity of the insula have been confirmed to be disturbed (39, 40), and gray matter volume also changed (41–43). The left insula is associated with empathy in affective perceptual and cognitive assessment forms (44). These studies reveal the vital role of the left anterior insula in social emotions, such as empathy, which also explains why patients with depression show higher personal pain and lower empathic attention to others when facing emotional situations.

The caudate was coactive with higher-level cognitive areas, such as the rostral anterior cingulate, dorsolateral prefrontal cortex, and inferior frontal gyri (45). The stimulation of the dorsolateral prefrontal cortex elevated neural activity and dopamine release in the caudate nucleus confirming it (46, 47). All these structures are well-known for their role in emotional and cognitive modulation and aberrations in the caudate nucleus in patients with MDD. For example, earlier studies found an abnormal increase in cerebral blood flow (GBF) in the right caudate of depression (48), and FC has a metabolic basis coupled with CBF and the rate of metabolism (49, 50). This indicates that FC in the right caudate is elevated in patients with MDD. In addition, Amiri found that the degree values of ventral caudate were substantially bilaterally higher in treatment-resistant depression (TRD) than in HCs (51). Those were the same as some of our results, indicating abnormalities in the caudate in patients with MDD. We found elevated DC in the left caudate and a decrease in the right caudate of MDD, which was reported for the first time in known studies. The possible explanation is that there are differences in bilateral cerebral hemispheres of MDD. Although this study is a cross-sectional design, we could not provide a reasonable explanation for this result; it presents a new idea for exploring the neural mechanism of MDD.

### Limitations

This study had several limitations. First, we did not determine the disease course of patients with MDD (current patients with MDD and remitted patients with MDD). In future studies, we would set stricter exclusion criteria to exclude remission-induced depression. Since this was a cross-sectional study, the structural changes caused by MDD could not be reflected based on the changes in the DC value, so it was necessary to use other calculation methods for further research. Finally, noise could not be eliminated. The patients were requested to keep quiet as much as possible to reduce the error caused by physiological noise.

## Conclusion

SVM combined with neuroimaging technology has been widely used in the study of various diseases. For example, Chen et al. found that combining the average ALFF and fall values of the right caudate nucleus and corpus callosum can diagnose MDD [accuracy (79.79%), sensitivity (65.12%), and specificity (92.16%)] (52). Gao et al. found that the combination of increased fALFF in the right precuneus and left superior frontal gyrus (SFG) with a diagnostic accuracy of 76.39% (18). When the sensitivity or specificity is less than 60%, this index may not meet the criteria of “diagnostic markers” (53). Our study found that DC values in the left antenna cerebellar lobe and right caudate could distinguish MDD from HCS with accuracy, sensitivity, and specificity of 87.71% (353/432), 84.85% (168/198), and 79.06% (185/234), respectively. To our knowledge, this study is the first to evaluate the utility of combining abnormal DC values in the left anterior cerebellar lobe and the right caudate nucleus as neuroimaging markers of MDD and provides a new idea for the diagnosis of MDD.

## Data availability statement

The raw data supporting the conclusions of this article will be made available by the authors, without undue reservation.

## Ethics statement

The studies involving human participants were reviewed and approved by the Ethics Committee of Tianyou Hospital Affiliated to Wuhan University of Science and Technology. The patients/participants provided their written informed consent to participate in this study. Written informed consent was obtained from the individual(s) for the publication of any potentially identifiable images or data included in this article.

## Author contributions

HL and XX conceived the project idea. YG implemented the method and performed the experiments. HR and SX supervised the project. JH provided critical suggestions for the design of the experiment. All authors contributed to the article and approved the submitted version.

## Footnotes

1
http://rfmri.org/dpabi

2
http://www.restfmri.net/

3
https://www.csie.ntu.edu.tw/~cjlin/
