# Supplementary material for: Abnormal degree centrality values as a potential imaging biomarker for major depressive disorder: A resting-state functional magnetic resonance imaging study and support vector machine analysis
Source: Front Psychiatry. 2022 Sep 6;13:960294. doi: 10.3389/fpsyt.2022.960294 (PMC9486164; doi:10.3389/fpsyt.2022.960294)
Supplement: Supplementary file 1 [file Data_Sheet_1.docx]

**Degree Centrality Analysis**

DC is a theory-based graph method to elucidate the connection degree between each node and other nodes in the network. The REST (http://www.restfmri.net/) software calculated the voxel-based DC value of the whole brain gray matter. To obtain each participant’s graph, Pearson correlation coefficients were computed between the time series of all pairs of brain voxels. As a result, we obtained an n × n matrix of Pearson correlation coefficients between any pair of voxels to construct the whole-brain FC matrix for each participant. Then, individual correlation matrices were transformed into a Z-score matrix using Fisher’s r-to-z transformation to improve normality. The weighted DC strength of a voxel as the sum of the connections (Z-values) between a given brain voxel and all other voxels was then computed. Furthermore, standardized weighted DC maps were acquired by subtracting the mean value, and then dividing by the standard deviation within the whole gray matter mask. Finally, the resulting DC maps were spatially smoothed with a 6-mm full width at half maximum (FWHM) Gaussian kernel.

For an undirected graph with g nodes, the degree centrality of node i is the total number of direct connections between i and other g-1 nodes, which is represented by a matrix as follows:

$$\mathrm{DC}_{i}=\sum_{J=1}^{g} x_{\mathrm{ij}}\left( i\neq j \right)$$

Where DC_i_ represents the degree centrality of node i, $\sum_{j=1}^{g} x_{ij}$ is used to calculate the number of direct connections between node i and other j(g-1) nodes (i ≠ j, excluding the connection between i and itself; that is, the value of the main diagonal can be ignored). The calculation of DC_i_ is simply to sum the cell values of the corresponding row or column of node i in the network matrix.

the following Fisher transformation formula was used to convert the correlation coefficient into a Z score, thus further improving the normality:

$$Z_{i}=\frac{\mathrm{DC}_{i}-\mathrm{mean}_{\mathrm{all}}}{\mathrm{std}_{\mathrm{all}}}$$

where Z_i_ is the Z score of the i^th^ voxel; DC_i_ is the DC value of the i^th^ voxel; mean_all_ is the average of all voxels in the brain structure; and std_all_ is the standard deviation.

**Classification Analysis**

SVM classification aims to find the maximum marginally separated hyperplane in a high-dimensional space for data classification. We use LibSVM method based on Weka. LibSVM is a library about the SVM developed by Professor Lin et al. in 2001. It has been widely used in bioinformatics, and thus has become the most widely used SVM Library in China. This library tool can be accessed at https://www.csie.ntu.edu.tw/~cjlin/. Weka is a free data mining platform that can meet various data analysis needs. LibSVM classification has been supported since Weka version 3.5. The specific process is as follows: The first is feature selection. To explore the differences in imaging characteristics between MDD patients and HCs, two-sample two-tailed t-tests were performed on all individual maps in a voxel-by-voxel manner. Voxels with P < 0.01 was used to determine the significant differences. The mean characteristic values of the clusters that had significant differences between MDD patients and HCs were extracted as the classification features. We finally got seven brain regions with significant differences in DC values. Secondly, the DC values used as the features were screened to the SVM model to establish the hyperplane. The kernel type applied in the study was the radial basis function (RBF) kernel. The best parameters including c (penalty coefficient) and g (gamma) were selected through the grid search method in the training dataset. Finally, optimal hyperplane developing from the training data was applied to a new testing dataset to check the classification performance. The “leave-one-out” cross-validation method was applied to produce the highest accuracy, sensitivity, and specificity. However, the results are not satisfactory. Then we paired the brain regions (a total of seven brain regions) with significant differences in DC values, input their average eigenvalues into the support vector machine at the same time (which is equivalent to forming a new index), verified again, selected the combination with the highest accuracy (with good sensitivity and specificity) as the result.
